# Diagnostic Accuracy of Frailty Screening Instruments Validated for Use among Older Adults Attending Emergency Departments: A Systematic Review and Meta-Analysis

**DOI:** 10.3390/ijerph20136280

**Published:** 2023-07-03

**Authors:** Elizabeth Moloney, Mark R. O’Donovan, Duygu Sezgin, Evelyn Flanagan, Keith McGrath, Suzanne Timmons, Rónán O’Caoimh

**Affiliations:** 1 HRB Clinical Research Facility, Mercy University Hospital, University College Cork, Grenville Place, T12 WE28 Cork, Ireland; e.moloney@muh.ie (E.M.); markrdon94@gmail.com (M.R.O.); e.flanagan@ucc.ie (E.F.); 2 Department of Geriatric Medicine, Mercy University Hospital, Grenville Place, T12 WE28 Cork, Ireland; kmcgrath@muh.ie (K.M.); s.timmons@ucc.ie (S.T.); 3 School of Nursing and Midwifery, University of Galway, H91 TK33 Galway, Ireland; duygu.sezgin@universityofgalway.ie; 4 Centre for Gerontology and Rehabilitation, University College Cork, St Finbarr’s Hospital, Douglas Road, T12 XH60 Cork, Ireland

**Keywords:** frailty, screening, tool, instrument, older adult, diagnostic test accuracy, emergency department, systematic review, meta-analysis

## Abstract

Early identification of frailty can prevent functional decline. Although multiple frailty screens exist for use in Emergency Departments (EDs), few are validated against diagnostic standards such as comprehensive geriatric assessment. To examine the diagnostic accuracy of ED screens for frailty, scientific databases were searched for prospective diagnostic accuracy test studies from January 2000 to September 2022. Studies were assessed for risk of bias using QUADAS-C. Psychometric properties were extracted and analysed using R. Six studies involving 1,663 participants describing seven frailty screening instruments (PRISMA-7, CFS, VIP, FRESH, BPQ, TRST, and ISAR), representing 13 unique data points, were included. The mean age of participants ranged from 76 to 86 years. The proportion that was female ranged from 45 to 60%. The pooled prevalence rate of frailty was high at 59%. The pooled estimate for sensitivity was 0.85 (95% CI: 0.76–0.91) versus 0.77 (95% CI: 0.62–0.88) for specificity. Pooled accuracy based on area under the ROC curve was 0.89 (95% CI: 0.86–0.90). Although few studies were found, limiting the ability to conduct a meta-analysis of individual instruments, available frailty screens can accurately diagnose frailty in older adults attending the ED. As specificity was comparatively low, additional assessment may be required to identify those requiring inpatient management or onward community referral. Further study is therefore required.

## 1. Introduction

Global population ageing is placing a heavy demand on healthcare systems across the world, especially in hospital emergency departments (EDs), where attendances of older adults with complex frailty syndromes have increased [1]. Over the next 35 years (2015–2050), the number of older people is expected to triple, and there will be a greater proportion living longer with chronic conditions [2,3]. High prevalence rates for frailty, an age-linked state of vulnerability [4], increase the risk of adverse outcomes in this population [4,5]. Identifying frail older adults should be a priority in acute medicine, given the adverse impact frailty can have on health outcomes and the high healthcare utilisation costs that ensue [6].

Although older adults represent 25% of those presenting to the ED [7], those with frailty account for up to 60% of older attendees [8]. Frail older patients have higher ED conversion rates [9] that are associated with prolonged hospital admissions [10], a higher chance of readmission, and a higher rate of inpatient mortality [10,11]. This cohort also constitutes rising numbers of unscheduled care admissions [11] to the perceived main access portal to acute care, the ED [12]. The challenge is that many EDs are not in a position to meet the care needs of frail older adults [13].

Two established frailty assessment methods exist: the Fried Frailty (physical) Phenotype model [14] and the cumulative deficit approach [15]. Both frailty models can predict outcomes in older adults in ED [8], but both have challenges to use in “real life” clinical practice. The Fried Frailty Phenotype requires physical assessments and accruing details that are often not feasible in an urgent care or ED environment, and it defines frailty in ordinal terms [14]. A frailty index, based on the accumulated burden of deficits, can be complex, time-consuming, and difficult to operationalize and take action on in the ED [10]. For example, it is estimated that 30 variables are optimal to generate a frailty index with good predictive validity for adverse outcomes in ED [12]. In tandem with growing awareness of ageing populations, new frailty screening instruments have been created that show an association between older adults with frailty and adverse events [16]. Such instruments are based on deficit accumulation indices or multi-domain physical decline. Instruments validated for use in acute care include the Identification of Seniors at Risk Tool (ISAR) [17], the Clinical Frailty Scale (CFS) [18], and the FRAIL scale [19]. All differ in terms of features, expertise required, and time needed for application. Although most instruments rate better than chance in predicting adverse events, most perform either poorly or very poorly [20,21].

Current obstacles that prevent early recognition of frailty in ED consist of overly complicated frailty pathways and proformas, with assessments that do not occur in real time with the person [13]. In fact, frailty status in the ED is often established after a decision about a patient’s care has been made [13]. Elliott highlighted only four peer-reviewed studies that related to frailty risk categorization instruments in ED [22]. Low rates of completion were recorded for all instruments (52%), with slow rates of completion recorded (1–10 min) [22]. Although a multitude of frailty screening instruments exist, few have been compared with a “gold standard” reference test such as a comprehensive geriatric assessment (CGA) [23] in what are known as diagnostic test accuracy (DTA) studies. Most studies instead examine predictive validity for adverse outcomes such as future healthcare use and mortality; further reporting in such studies is not optimal and requires better standardisation [24]. This systematic review and meta-analysis therefore seeks to synthesise the existing evidence concerning the diagnostic accuracy of current frailty screening instruments used in EDs to identify frailty in older adults. Understanding this will facilitate recommendations on the best available short screen for use in ED and have implications for guidelines in this setting [25].

### 1.1. Study Design

A systematic review was undertaken to identify research publications that validated frailty screening instruments among older adults in the ED. It conformed to the recommendations laid out in the Cochrane Handbook for Systematic Reviews of Diagnostic Test Accuracy [26] and the Preferred Reporting Items for Systematic Reviews and Meta-Analyses Protocols (PRISMA) standardised reporting recommendations [27]. The Preferred Reporting Items for Systematic Reviews and Meta-Analyses Protocols (PRISMA-P) guidelines were adhered to in fulfilling study protocol requirements [28]. The steps taken to produce this systematic review included: (1) clearly stating the research question; (2) recording the protocol on the global register of systematic reviews (PROSPERO number CRD42020216780); (3) conducting database searches; (4) selecting suitable studies as per the inclusion criteria; (5) selecting data from included studies; and (6) amalgamating the results.

### 1.2. Eligibility Criteria

Studies were chosen based on population, index test, reference test, and diagnosis of interest (PIRD) criteria for diagnostic test accuracy reviews [29]. Details are illustrated in Table 1. The population included older adults aged 60 years and over screened in ED with any available frailty screening instrument (index test). Comparators (reference standards) included the “gold standard” CGA or deficit accumulation index (DAI) frailty model used to diagnose frailty (diagnosis of interest). Descriptive studies, observational studies, and randomised controlled studies were included where frailty screening instruments were utilised to screen older adults and measured against the described reference test to assess the diagnostic accuracy of the instrument.

### 1.3. Exclusion Criteria

Studies were excluded if the study population mean or median age was <60 years or where data could not be extracted separately on those aged ≥ 60 years. Access to full-text articles was required. Case reports or series, commentary pieces, opinion articles, conference abstracts, editorials, protocol submissions, and review articles were rejected; however, searches of the reference lists of relevant review papers for additional studies were included in the review. Authors of such papers, including abstracts, were contacted to inquire if such papers had been submitted and if these data could be obtained in advance of publication for potential inclusion. If a frailty screening instrument was used in a validation study of another instrument, it was excluded, and research articles that reported on frailty but omitted using or naming a specific instrument were also rejected. Studies where an instrument was used in a specific population or condition (e.g., chronic kidney disease, malignancy, heart failure) were also excluded.

### 1.4. Information Sources (Search Strategy)

The preliminary database query incorporated publications published from 1 January 2000 to 20 March 2021. These computer searches were updated on 30 November 2021 and 1 September 2022 in advance of submission. The search was limited to studies published after 2000, as prior to this, frailty was not a widely used clinical research term; for example, no publications including the terms “frailty” AND “emergency department” were found in a search of PubMed prior to 2004. The electronic databases searched were PubMed, Cinahl, Cochrane, Embase, Google Scholar, and TRIP. The complete search strategy is shown in Appendix A. Studies in all languages were included and translated where required. All searches were imported into the Endnote citation management tool, with duplicate citations eliminated.

### 1.5. Study Selection and Data Retrieval

Study titles and abstract content were independently reviewed based on the requirements for inclusion by two researchers (EM, DS). Studies meeting the required criteria were reviewed in full, and final eligibility for inclusion was assessed by the principal investigator (RO’C). Any disagreements were managed by consensus. Data were retrieved from accepted studies by two independent researchers (EM, DS) using standardised proformas to collect the following data, as advised by Campbell et al. (2015) [30]:

- Administrative details: Author, title, type of publication, journal, country.
- Study features: Design, aim(s), sample size, demographics, clinical location, results.
- Components of the instrument(s) used to measure frailty: number of domains/items, what are the domains/items, whether it requires specialised equipment, mode of administration, administration time, details on the development of the tool, and measurement properties.
- Which “gold standard” the instruments being studied were compared with (e.g., CGA or other frailty measures).
- Prevalence of frailty measured.

Disagreement regarding data retrieval was resolved by consensus. If not directly reported, sensitivity, specificity, positive predictive value, and negative predictive value were calculated based on study findings or (in the case of insufficient data) on additional information obtained from the authors. Missing or incomplete data were dealt with by contacting the study author(s). Missing values were recorded in the data extraction form.

### 1.6. Risk of Bias

The methodological standards of the accepted publications were evaluated independently by two researchers (EM and RO’C) using the Quality Assessment of Diagnostic Accuracy Studies-Comparative (QUADAS-C) tool, an extension of the QUADAS 2 tool to assess the risk of bias and diagnostic accuracy in comparative studies [31]. Disagreements were resolved by a third reviewer (KM).

### 1.7. Analysis

The analysis was performed using R version 4.2.0. Forest plots of the sensitivity and specificity of each of the instruments were generated with a 95% confidence interval (CI) for each estimate. A bivariate random-effects model proposed by Reitsma et al. (2005) [32] was used to calculate pooled sensitivity and FPR (1-specificity) from the True Positive (TP), False Negative (FN), False Positive (FP), and True Negative (TN) of each instrument. Pooled prevalence was calculated from the data by taking the sum of (TP+FN)/(TP+FN+TN+FP). Youden’s Index [33]: positive likelihood ratio (PLR), negative likelihood ratio (NLR), positive predictive value (PPV), negative predictive value (NPV), and accuracy were also calculated using the bivariate random effects model. A summary receiver operating characteristic (ROC) curve (SROC) was graphically displayed for the bivariate random effects model, and the pooled area under the curve (AUC) of the SROC curve was calculated. A 95% CI was generated for this AUC estimate using a parametric bootstrap with 2000 resamples. Descriptive text was used where combined data pooling was not feasible (i.e., less than three data points were available).

## 2. Results

### 2.1. Search Results

**Figure 1:**
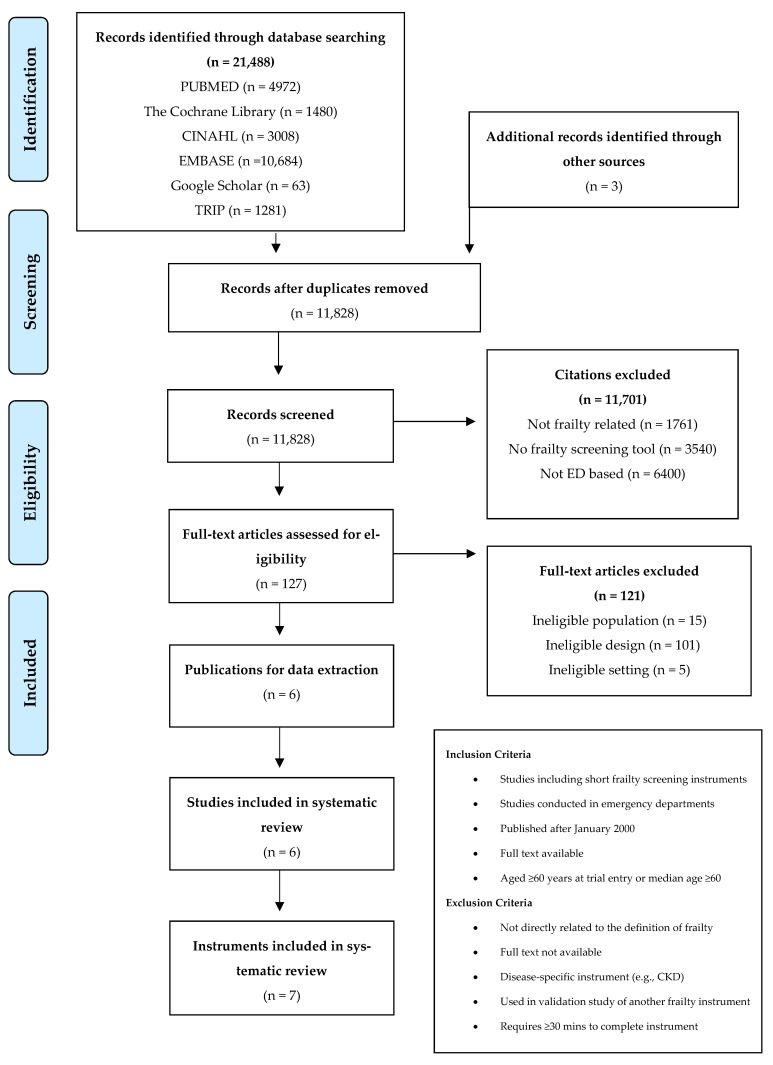
PRISMA Flow Diagram illustrating selection process for included studies.

Database searches returned 21,488 citations. Duplicate records were discarded, and extra hand search results added an additional three papers to give a total of 11,828 citations to the screen. Of these, 11,701 were removed based on title description and abstract content, leaving 127 citations for full text review. A detailed review of these 127 articles led to the rejection of 120 studies for the following reasons: ineligible design (n = 100), ineligible population (n = 15), or ineligible setting (n = 5). A PRISMA flow diagram illustrates the selection details in Figure 1. Accordingly, six studies [8,10,34,35,36,37,38] describing seven frailty screening instruments were included in this systematic review. Some papers included more than one of these instruments, yielding 13 unique data points. Additional details were requested from the authors of one relevant paper, initially found as an abstract publication [37], as the full-text article was unpublished at the time of the literature search (now published [38]). A table providing additional details of the search strategy utilised is included in Appendix A.

### 2.2. Quality of Methodology of Included Publications

Table 2 illustrates the quality of the included studies according to the QUADAS-C assessment instrument. Reviewer agreement was 90% on all aspects of the quality assessment. Regarding patient selection, the risk of bias was generally considered low, and there were no concerns about the applicability of the selection criteria. Three studies [8,34,35] did not specify whether the results of the frailty screening instrument (index test) and CGA (reference test) were independently scored, and so the risk of bias was unclear in those circumstances. However, the flow and timing of these studies raised few concerns.

### 2.3. Study Characteristics

All studies were prospective in design. Consecutive sampling was documented in the methods section of four papers [10,34,36,38]. The seven studies included 1663 participants. The mean sample size was 257 patients, ranging from 161 [34] to 498 [36]. The included studies were conducted in Ireland (n = 2) [10,37,38], Italy (n = 1) [8], France (n = 1) [35], Sweden (n = 1) [34], and Canada (n = 1) [36]. All studies were published in English. The majority of studies (n = 6) were published in the latter half of the 2010s [10,34,35,36,37,38]. The age as an inclusion criterion varied from ≥65 years [8] to ≥75 years [36]. The reported mean age of study participants was 79.8 years (range 76–86); 52% were female (range 45–60%). All six studies used CGA as a “gold standard” for measuring frailty. The pooled prevalence rate of frailty was high at 59%.

The instruments included different frailty domains and questions, varying in number from one to nine. Regarding the domains included, all seven instruments evaluated physical deficits such as “slowing up”, loss of activity levels, ability to mobilise, activities of daily living (ADLs), instrumental activities of daily living (IADLs), physical function, falls, communication (vision and hearing), hospitalisation, and excess or inappropriate prescribing (i.e., polypharmacy). Two instruments (the ISAR and BPQ) were screened for both physical and cognitive aspects (memory impairment), and three (the E-TRST, VIP, and PRISMA 7) were screened for both physical and social aspects (social support). A detailed description of all the included studies and frailty indicators is included in Appendix A.

Study authors were contacted regarding administration time if clarification was needed from the research article. Some of the instruments were ultra-short to administer (<2 min), such as the Bergman Paris Question (BPQ) [35] and Variable Indicator of Risk Placement (VIP) [10,37,38], while others were performed in more than one stage, such as the Triage Risk Screening Tool (TRST) [36], with its initial screen, the Emergency-TRST (E-TRST), usually taking less than 2 min [39]. From previous studies in ED, usual administration times for the ISAR vary from 2 to 5 min [40], while the FRESH instrument is recorded as taking ≤5 min [34]. The time for the CFS is more difficult to assess, as ideally, it should not be completed until a more detailed assessment has been completed; nevertheless, an ultra-short time of 24 s has been previously recorded in ED when the CFS is used as a stand-alone screen [41]. The documentation of administration times was variable. Only one study documented an administration time for the completion of all the instruments examined in their study. All the first authors of these studies were contacted regarding the administration times of the screens. Responses were received from three authors: Kajsa (2016) [34] for the FRESH instrument, Lague (2018) [35] for the BPQ instrument, and Salvi (2012) [8] for the ISAR instrument, who confirmed that no timings were taken during the studies and only estimates of the administration times for the screens in those studies could be provided. All three authors reported estimated administration times of between 2 and 5 min to complete these three screens. The highest AUC for diagnosing frailty was reported for the ISAR [8] at 0.92 (95% CI: 0.88–0.96) and the lowest for the BPQ [35] at 0.63 (95% CI: 0.53–0.72). Study characteristics are summarised in Table 3.

### 2.4. Meta-Analysis

#### Pooled Estimates for Frailty

In total, seven papers reported on the diagnostic accuracy of frailty screening instruments in ED [8,10,34,35,36,37,38]. Four of these reported results for more than one frailty screening instrument or version thereof, leading to a total of 13 datasets. The numbers TP, FP, FN, and TN were retrieved from the papers, and where unavailable, they were calculated from the sensitivity, specificity, and frailty prevalence values. The sensitivities and specificities for each of the 13 datasets are displayed in Table 4.

Examining the instruments individually in Table 4, the sensitivity values for detecting frailty based on CGA varied widely. The PRISMA-7 was reported as the most sensitive, with a sensitivity ranging between 0.84 and 0.98 but with a moderate specificity of 0.64–0.78. The CFS was the least sensitive instrument but most specific, suggesting it may need to be combined with another frailty instrument that is more sensitive for diagnosing frailty [10]. The ISAR showed high sensitivity, ranging between 0.94 and 0.95, but poor specificity in both studies (0.35–0.63). The geriatrician-assisted TRST (G-TRST) was more sensitive than the ED physician version (E-TRST) (0.93 versus 0.88). There was little difference between the FRESH 4-item and FRESH 5-item screens in terms of sensitivity (0.84 versus 0.81) or specificity (0.75 versus 0.80) [34]. The BPQ had high sensitivity (0.94) but very poor specificity (0.29) [35]. Given these findings, there were insufficient data available to conduct a meta-analysis of the individual instruments. Pooled estimates of diagnostic accuracy data for frailty are presented in Table 5.

**Figure 2:**
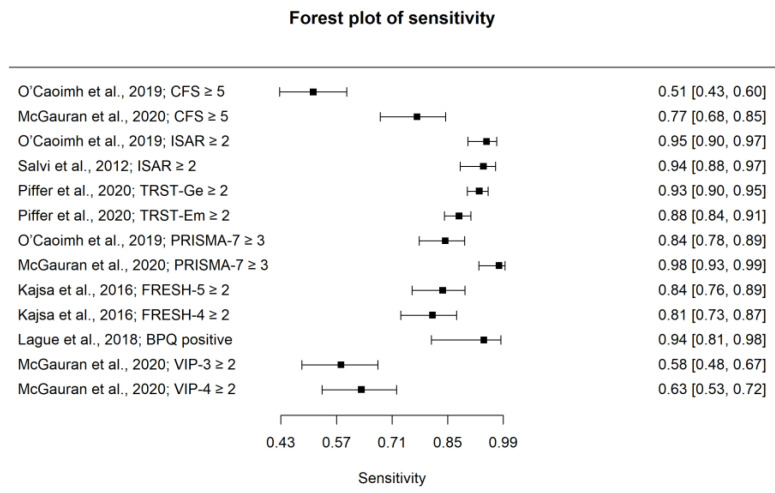
Forest Plot of sensitivity for detecting frailty.

**Figure 3:**
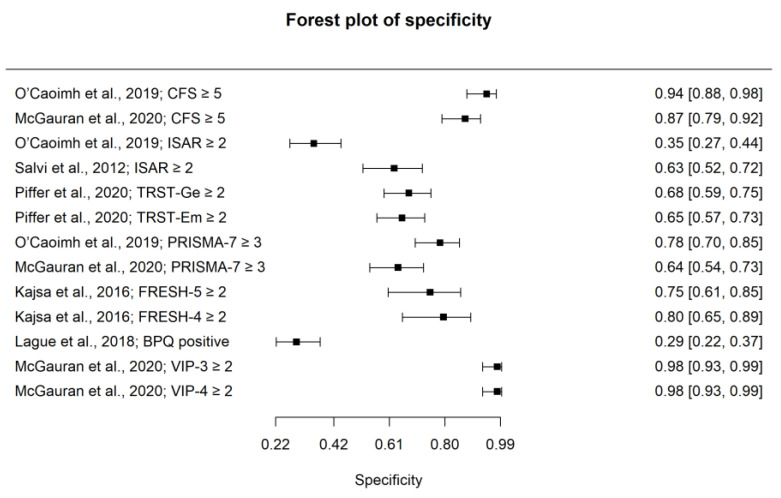
Forest Plot of specificity for detecting frailty.

**Figure 4:**
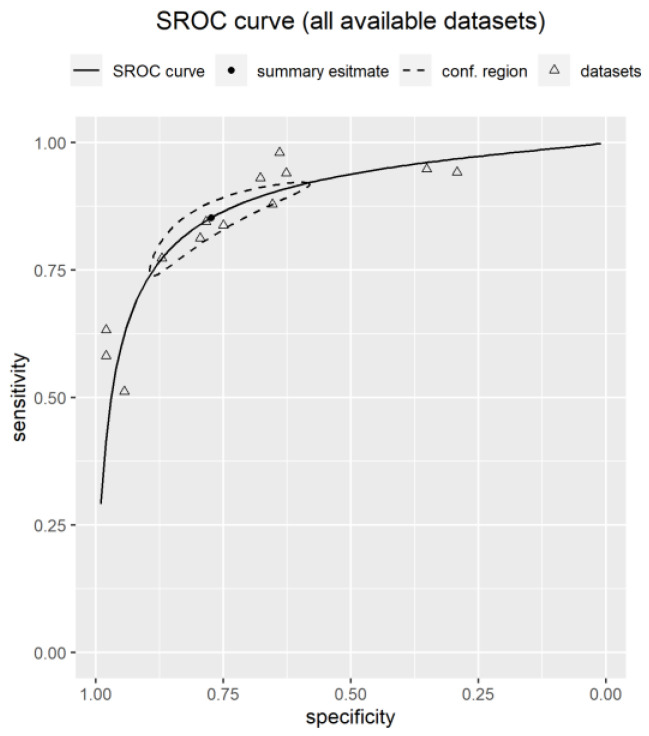
SROC curve for the meta-analysis of all available data (n = 13 data points). The summary estimate is the pooled sensitivity/specificity estimate from the bivariate random effects model and the conf. region is a 95% confidence region for this summary estimate.

The pooled estimate for sensitivity for detecting frailty for all instruments included in the meta-analysis was 0.85 (95% CI: 0.76–0.91), with a pooled estimate for specificity of 0.77 (95% CI: 0.62–0.88). The PPV was 0.85 (95% CI: 0.79–0.91), and the NPV was 0.77 (95% CI: 0.7–0.80). The pooled estimate for accuracy based on the AUC was 0.89 (95% CI: 0.86–0.90). The pooled PLR for frailty was 3.91 (95% CI: 2.41–6.23) with a NLR of 0.2 (95% CI: 0.14–0.27). Summary forest plots for sensitivity and specificity are displayed in Figure 2 and Figure 3, respectively. An SROC curve, a visual representation of the overall diagnostic accuracy of pooled data for the meta-analysis of all available datasets (n = 13), is displayed in Figure 4. The 13 triangles represent each data point, and the black line is the SROC curve.

## 3. Discussion

### 3.1. Overview

This systematic review and meta-analysis examined the diagnostic accuracy of available short frailty screening instruments as determined by an independently conducted assessment of frailty (i.e., DTA studies conducted blind to the scores of the screens). In all, only seven different frailty screening instruments across just six studies were found. Most were not bespoke frailty screens. The TRST, VIP, and ISAR were instead designed as risk-prediction instruments for use in acute care settings such as the ED with a view to predicting the risk of adverse outcomes rather than measuring frailty, which was not screened for when these instruments were first developed. Multiple reviews have shown that such individual instruments have reasonable-to-good predictive validity for future adverse health outcomes among older patients attending the ED, including mortality [42], re-admission [42,43], and prolonged length of hospital stay [44]. This is the first systematic review, to our knowledge, that confirms that they are actually accurate in identifying frail older people in ED who may require more detailed assessment and tailored management.

Based on the data obtained in the systematic review, diagnostic accuracy and properties such as sensitivity and specificity for frailty varied markedly between studies and individual instruments. In particular, the c-statistic (AUC) values for the instruments published varied from 0.63 for the BPQ instrument to 0.95 for the CFS instrument. However, a pooled meta-analysis showed that overall, these instruments had good to excellent diagnostic accuracy with high levels of sensitivity and specificity for frailty, as measured against the CGA, usually regarded as the “gold standard” for measuring frailty [45]. The pooled estimate for PPV for all instruments was also good (85%), confirming that these are useful as screening tests with a high likelihood that they are detecting true frailty. A high PPV is an important characteristic of a screening test [46]. The overall pooled diagnostic accuracy measured using AUC was 0.89, indicating good accuracy. There was insufficient data available to conduct a meta-analysis of the individual instruments. Hence, it is difficult to recommend any single instrument based on this. Based on the data found in the review, the PRISMA-7 had the highest sensitivity and the CFS the highest specificity.

The PRISMA-7, although originally advised by the British Geriatrics Society as the instrument to opportunistically screen older people for frailty in different settings [47] is less studied in ED, though a dedicated systematic review is planned to clarify further [48], The CFS is one of the most widely used instruments, with growing evidence for predictive validity for adverse outcomes in large samples [42,43,49]. However, a recent scoping review of the use of the CFS showed that reporting is not optimal and more standardisation of results is needed to better interpret its utility as a screen for frailty in ED [24]. In addition, its administration time is ill-defined, particularly given that many suggest that the CFS should only be conducted after a CGA when information is gathered to stratify a patient adequately [50]. Of the other instruments, the diagnostic accuracy of the ISAR was only studied in two papers [8,10]; in these studies, it showed high sensitivity but only low-moderate specificity. The ISAR is one of the most widely utilised screening instruments to highlight older persons at risk of functional deterioration in ED, but Galvin et al. (2017) [17] have previously recommended further evaluation of the ISAR rule owing to variations in patient and process outcomes in a systematic review evaluating adverse outcomes of older adults attending ED [17]. It is used to identify frailty but is less accurate than other instruments. Only one study examined the VIP, comparing 3 and 4-item versions [37,38]. The VIP 4-item version had low sensitivity, high specificity, and low false positives [37,38]. Although it is a quick and easy-to-use tool that does not require much training, it predominantly identifies people who are more severely frail. Further evaluation of the validity and reliability of the VIP as a frailty identification tool is recommended [51].

### 3.2. Results in Context

The diagnostic accuracy of frailty screening instruments in older adults has previously been reported in an umbrella review by Apóstolo et al., (2017) [43]. Using baseline results from the Cardiovascular Health Study (CHS) phenotype model, the Canadian Study of Health and Ageing (CSHA) DAI model, and the frailty index based on a CGA (FI-CGA), short frailty assessments in all settings were examined [43]. The PRISMA-7 was the only screening instrument from our study to be included in this umbrella review, which confirmed high sensitivity and specificity to identify frailty in adults over 65 years. However, no information relating to DTA studies in ED was reported. Apóstolo did note that only a few frailty instruments reviewed seemed to be diagnostically accurate, but there was clear usefulness to using simple risk indicators such as gait speed [43]. Our study differs from existing publications in that it examines DTA studies in the ED setting, a clinical area that lacks previous systematic review publications. To illustrate this, one only needs to review the two most up-to-date frailty evaluation study protocols, which omit the ED as a study location [48,52]. Our study fills an important data vacuum in the ED environment, aligning with EUGMS geriatric emergency medicine service recommendations to consider the use of frailty screening instruments in the ED [6]. The COVID-19 pandemic has shown that this is timely by highlighting the importance of early recognition of frailty as a way to improve the triage of older patients and identify those most likely to benefit from critical and intensive care [53]. The difficulty of staff without geriatric medicine training using frailty screening instruments has also been shown to be challenging [54]. Inaccurate and poorly understood frailty assessments can lead to inadvertent changes to treatment access, while decisions based solely on chronological age or current presentation have concerned older adult advocates worldwide [53,55]. Without clear guidelines on the most appropriate frailty screening tool to use in the ED environment and subsequent training of ED staff, the integration of routine frailty screening into Geriatric Emergency Medicine protocols will be hindered [56].

Lengthy administration time is often identified as a key barrier to the use of frailty screening tools by ED staff, with some tools requiring 30–40 min to complete. Administration times were not clearly documented in any of the studies included in this systematic review. These data were acquired by contacting the study authors, who could only give estimates of completion times for three of the screens. While it was previously reported in another systematic review that similar frailty screening tools have administration times of less than five minutes [22], this would appear to relate predominantly to the ISAR tool. In order to integrate frailty screening into routine ED practice, it is important that the screening process be shown to be feasible so as to be implemented in a busy clinical environment, and hence the rapid application times of frailty screening tools should be clearly highlighted. Thus, we recommend that future studies include mean/median administration times to help clinicians decide which is the optimal tool for their own practice.

### 3.3. Strengths and Weaknesses

Positive strengths of this work include the comprehensive search strategy that followed Cochrane procedures with no limitation on language, as well as the manual search of references in the included studies. In addition to contacting authors, the research team was able to calculate some missing values based on the data provided. The UN older person classification of older adults as those aged ≥ 60 years was used to avoid limiting the external validity of the studies [57]. As far as we are aware, this is the first review to combine the accrued evidence on the diagnostic accuracy of current frailty screening instruments used in EDs to identify frailty in older adults. 

Despite attention being paid by the research team, a risk remains that all appropriate studies may not have been included in maintaining methodological rigour. For example, since the search was completed, the interRAI Emergency Department Screener has been validated against the interRAI Emergency Department Assessment System to identify those experiencing complex health and social concerns and was found to have a mean administration time of <2 min and good sensitivity and specificity for this outcome [58]. While that study did not include frailty as a specified outcome, it identified those in need of CGA, which could be taken as a surrogate for frailty. From the six studies, including seven index tests, four had relatively small numbers of participants and all represented patients recruited from high-income western countries (Europe and Canada) [8,34,35,37,38], which could limit the generalizability of the results. The pooled prevalence of frailty across the studies included was 59%, which is relatively high and suggests that these studies were at risk of spectrum bias. The data were markedly heterogeneous, and the small sample size of studies available for most instruments limited the ability to perform a meta-analysis. Instruments that did not have sufficient numbers to enable this may be more accurate, but this review could not confirm this, suggesting the need for additional studies. Finally, as the search was widened to include additional databases (e.g., TRIP) and was updated on two occasions due to delays with the submission, there is discordance with the published PROSPERO protocol.

### 3.4. Clinical Implications and Future Research

While there may be a multitude of frailty screening tools currently available, this systematic review and meta-analysis has shown that utilising a two-step assessment process with an index diagnostic screening test and gold standard reference test model allows accurate frailty diagnosis in the ED. It is argued that this may not be feasible in routine practice, but our results reinforce the view of Heeren et al. that incorporating more objectively validated measures into future vulnerability studies may improve instrument accuracy [59]. This would also align with the theory that if risk stratification for common geriatric syndromes is embedded at the beginning of the ED process, and can be part of classic nurse triage or doctor assessment at triage, which has shown improved ED performance [60]. From this research, it is clear that the TRST, VIP, and ISAR were not originally designed as frailty screening instruments but as risk-prediction instruments, with frailty screening retrospectively considered as an additional measurement component.

In a clinical context, this suggests that efforts should be made to improve the DTA of validated short frailty screening tools using this two-stage assessment process, where feasible in ED environments, and apply frailty screening in regions of high frailty prevalence, such as sub-Saharan Africa. Scant attention has been paid to ageing populations in low- and middle-income countries, which is concerning given the rapid population growth of older adults in these regions, where life expectancy has also improved [61]. Accurate frailty identification in EDs is a rapid mechanism that selects older adults for adapted trajectories to deliver the “*right care to the right person at the right time and place*” [62]. Such frailty data should assist public health strategic planning in areas under increasing strain, as highlighted by the WHO Study on Global Ageing and Adult Health Report [63].

## 4. Conclusions

Overall, although a limited number of heterogenous studies and instruments were available for review, the result of this pooled DTA meta-analysis suggests that the available short screens for frailty had good sensitivity, reasonable specificity, and good to excellent accuracy as measured against CGA. The PRISMA-7 was the most sensitive instrument for identifying frailty, while the CFS had the highest specificity, albeit with insufficient data to conduct a meta-analysis of individual instruments. Further high-quality studies utilising an independent gold standard such as the CGA are required before a specific instrument could be recommended. Despite the relative paucity of studies included, no major methodological concerns were noted. Our work demonstrates that while the use of these short frailty screens in ED appears to be accurate in diagnosing frailty, more research is now required comparing additional instruments in large samples and in different populations and settings to properly examine the psychometric performance of individual instruments among older adults attending ED.

## Figures and Tables

**Table 1:** Question layout for this systematic review using the population, index test, reference test, and diagnosis of interest (PIRD) system for diagnostic accuracy studies.

PIRD Question Layout			
Population	Index Test	Reference Test	Diagnosis of Interest
Persons aged ≥ 60 years presenting to ED utilising an established definition of frailty	Short screens and risk stratifying instruments utilised to highlight older adults with frailty	CGA or components of existing frailty models in the context of independent review	Accurate frailty diagnosis and prediction of defined adverse events

**Table 2:** Quality assessment of included studies (n = 6) in the review using the Quality Assessment of Diagnostic Accuracy Studies-Comparative (QUADAS-C).

Study	Risk of Bias				Applicability Concerns		
	Patient Selection	Index Test	Reference Standard	Flow and Timing	Patient Selection	Index Test	Reference Standard
Salvi et al., 2012 [8]	Low	Low	Unclear	Low	Low	Low	Low
Kajsa et al., 2016 [34]	Low	Unclear	Unclear	Low	Low	Low	Low
Lague et al., 2018 [35]	Unclear	Unclear	Low	Low	Low	Low	Low
O’Caoimh et al., 2019 [10]	Low	Low	Low	Low	Low	Low	Low
McGuaran et al., 2020 [37]	Low	Low	Unclear	Low	Low	Low	Low
Piffer et al., 2020 [36]	Low	Unclear	Unclear	Low	Low	Low	Low

**Table 3:** Features of included studies in the systematic review of diagnostic accuracy tests for frailty in the emergency department.

Study (Country)	N = X	Mean or Median Age	% F	Inclusion Criteria	Study Design	Frailty Screens Used with Cut-Offs	Reference Standard	Time to Complete	AUC for Each Screen (95% Confidence Interval)
Salvi et al., 2012 [8], (Italy)	200	80	57%	≥65 years screened after triage	Prospective observation cohort, unclear sampling type	ISAR ≥ 2	CGA	ND	ISAR = 0.92 (0.88–0.96)
Kajsa et al., 2016 [34], (Sweden)	161	82	55%	≥80 years or 65–79 with ≥1 chronic disease and help ≥1 ADL	Cross-sectional, consecutive sampling	FRESH ≥ 2	CGA	ND	FRESH = 0.86 (0.79–0.92)
Lague et al., 2018 [35], (Canada)	171	76.9	52%	≥65 yrs Independent or semi-independent; ≥ 8 h in ED; Admitted to any hospital ward.	Prospective observational cohort, unclear sampling type	BPQ No= Positive	CGA	ND	BPQ = 0.63 (0.53–0.72)
O’Caoimh et al. 2019 [10], (Ireland)	265	78	54%	≥70 years screened at triage	Prospective cross-sectional, consecutive sampling	CFS ≥ 5 PRISMA-7 ≥ 3 ISAR ≥ 2	CGA	ND	CFS = 0.83 (0.77–0.88) PRISMA-7 = 0.88 (0.83–0.93) ISAR = 0.78 (0.71–0.84)
McGuaran et al. 2020 [37], (Ireland)	197	79	45%	≥70 years screened at triage	Prospective cross-sectional, consecutive sampling	VIP-3 ≥ 2 VIP-4 ≥ 3 CFS ≥ 5 PRISMA-7 ≥ 3	CGA	ND	CFS = 0.91 (0.87–0.95) PRISMA-7 = 0.91 (0.86–0.95) VIP-3 = 0.84 (0.78–0.89) VIP-4 = 0.84 (0.79–0.90)
Piffer et al., 2020 [36], (France)	498	86	60%	≥75-year-olds who (1). required hospital admission over 1 year; (2). were scored with the E-TRST, G-TRST, and SEGA within 24 h.	Prospective, cross-sectional, consecutive sampling	TRST ≥ 2	CGA	ND	E-TRST = 0.86 (0.83–0.89) G-TRST = 0.90 (0.87–0.93)

ADL: Activities of Daily Living; AUC: Area Under the Curve; BPQ: Bergman-Paris Question; CGA: Comprehensive Geriatric Assessment; CFS: Clinical Frailty Scale; ED: Emergency Department; F: Female; ISAR: Identification of Seniors At Risk; PRISMA-7: Programme of Research on Integration of Services for the Maintenance of Autonomy; VIP: Variable Indicator of Placement Risk; E-TRST: Emergency Physician administered TRST; G-TRST: Geriatrician administered TRST; ND = not documented.

**Table 4:** Performance of the eight screening instruments with cut-offs for identifying frailty including sensitivity (sens), specificity (spec).

Author	Year	Tool	TP	FN	FP	TN	N	Sen	Spec	Wt-Sen	Wt-Spec
O’Caoimh et al.,	2019 [10]	CFS ≥ 5	67	64	5	84	220	0.51	0.94	5.551	6.218
McGauran et al.,	2020 [37]	CFS ≥ 5	75	22	13	86	196	0.77	0.87	5.88	6.131
Salvi et al.,	2012 [8]	ISAR ≥ 2	110	7	31	52	200	0.94	0.63	6.075	5.457
O’Caoimh et al.,	2019 [10]	ISAR ≥ 2	146	8	72	39	265	0.95	0.35	6.105	5.342
O’Caoimh et al.,	2019 [10]	PRISMA-7 ≥ 3	130	24	24	87	265	0.84	0.78	6.065	6.061
McGauran et al.,	2020 [37]	PRISMA-7 ≥ 3	95	2	36	63	196	0.98	0.64	6.051	5.423
McGauran et al.,	2020 [37]	VIP-3 item ≥ 2	57	40	2	97	196	0.59	0.98	5.154	6.24
McGauran et al.,	2020 [37]	VIP-4 item. ≥ 3	61	36	2	97	196	0.63	0.98	5.263	6.23
Piffer et al.,	2020 [36]	G-TRST ≥ 2	345	26	41	86	498	0.93	0.68	6.16	5.822
Piffer et al.,	2020 [36]	E-TRST ≥ 2	326	45	44	83	498	0.88	0.65	6.16	6.025
Kajsa et al.,	2016 [34]	FRESH 4 item	98	19	11	33	161	0.84	0.75	6.03	5.486
Kajsa et al.,	2016 [34]	FRESH 5 item	95	22	9	35	161	0.81	0.80	6.001	5.591
Lague et al.,	2018 [35]	BPQ “No”	32	2	97	40	171	0.94	0.29	5.741	5.339

BPQ: Bergman-Paris Question; CFS: Clinical Frailty Scale; ISAR: Identification of Seniors At Risk; PRISMA-7: Programme of Research to Integrate the Services for the Maintenance of Autonomy 7; VIP: Variable Indicator of Placement; TRST: Triage Risk Screening Tool; E-TRST: Emergency Physician TRST; G-TRST: Geriatrician TRST. N = number; TP = True Positive; TN = True Negative; FP = False Positive; TN = True Negative; Sen = Sensitivity; Spec = Specificity; Wt-Sen = Weighted Sensitivity; Wt-Spec = Weighted Specificity

**Table 5:** Pooled diagnostic accuracy estimates with 95% confidence intervals (CI) calculated using a bivariate random-effects model [34].

Pooled Estimates (Datasets)	AUC (95% CI)	FPR (95% CI)	FNR (95% CI)	Sensitivity (95% CI)	Specificity (95% CI)	Youden’s (95% CI)	Accuracy (95% CI)	PPV (95% CI)	NPV (95% CI)	PLR (95% CI)	NLR (95% CI)
All available data points (n = 13)	0.89 (0.86–0.90)	0.23 (0.12–0.38)	0.15 (0.09–0.24)	0.85 (0.76– 0.91)	0.77 (0.62– 0.88)	0.62 (0.53– 0.67)	0.82 (0.79– 0.84)	0.85 (0.79–0.91)	0.77 (0.70–0.82)	3.91 (2.41–6.23)	0.20 (0.14–0.27)

AUC: Area Under the Curve; FPR: False Positive Ratio; FNR: False Negative Ratio; PPV: Positive Predictive Value; NPV: Negative Predictive Value; PLR: Positive Likelihood Ratio; NLR: Negative Likelihood Ratio.

## Data Availability

On request from the authors.

## Appendix A

### Description of Included Studies

The first study by Salvi et al. (2012) [8] was a sub-analysis of a prospective study of 200 adults aged ≥ 65 years who attended two urban Italian EDs over a four-week period. The unique feature of this study was to compare frailty determined by the Deficit Accumulation Index (DAI) aspect of a CGA with the ability of the ISAR screening instrument to discriminate frail from non-frail patients. Adverse outcome parameters at one-month and six-month intervals were collected on emergent ED attendances, frequent ED return (three or more emergent ED visits in six months), inpatient admission (less than six months post discharge), six-month functional deterioration (interpreted as inability to undertake ≥1 activity of daily living (ADL)), and 6-month mortality. Trained research assistants (RA) screened ED patients just after triage, with the aid of a family member if patient cognition was impaired. Elements included in the DAI assessment were the Charlson Co-Morbidity Index (CCI), the Short Portable Mental Status Questionnaire (SPMSQ) for cognition, and the Katz ADL for functional status baseline.

The second study by Kajsa et al. (2016) [34] assessed the DTA of the FRESH screening instrument in frailty identification among 161 Swedish adults aged ≥ 65 years who attended an emergency department over an 18-month study period. The FRESH screen consists of five brief questions. Four out of five questions concern the ability to mobilise, lethargy, fatigue, concerns regarding falling, and being dependent on others for shopping. The final question relates to frequent ED reattendances (≥3 in the last 12 months). Frailty status was established via the Fried Phenotype (FPH) with the addition of visual and cognitive impairment. Adults enrolled in the study were screened consecutively Monday–Friday in the ED (n = 144) by nursing staff with geriatric medicine training. Older adults presenting outside these hours were recruited on an inpatient ward or by study correspondence if discharged before being identified as potential participants (n = 17).

The third study by Lague et al. (2018) [35] was a sub-analysis of the INDEED multicenter prospective study. This involved 171 adults aged over 65 who required inpatient admission after a period in the ED (more than 8 h). The study enrolled participants in four Canadian EDs over a two-month period. Study objectives were to examine the predictive capacity of the single-line Bergman Paris Question (BPQ) as a screening instrument for three geriatric syndromes (cognition, function, and frailty) in independent or semi-independent older adults in the ED. A trained research assistant asked caregivers the BPQ: “Would you be comfortable leaving your family member home alone for three months if you had to go on a trip to Paris and no other family member or close friend was available?”.

Caregivers who indicated they would NOT be happy to leave their relative alone constituted a “positive BPQ,”, which pointed to a potential frailty syndrome. If the caregiver indicated they would be happy to leave their relative alone, this constituted a “negative BPQ”, which pointed to that relative not requiring specialist geriatric medicine input. Cognition was evaluated using the Telephone Interview for Cognitive Status-modified (TICS-m), functional ability via the Older American Resources and Services scale (OARS), frailty status was assessed using the Clinical Frailty Scale, and the Confusion Assessment Method (CAM) was utilised to rule out delirium. Data on sociodemographic profile, medication use, co-morbidity burden, and psychological status were also collected in the ED.

The fourth study by O’Caoimh et al. (2019) [10] compared the clinimetric characteristics of three frailty screening instruments used to identify frailty of the ED in an Irish tertiary care hospital. The three instruments were the Clinical Frailty Scale (CFS), Identification of Seniors at Risk (ISAR), and the Programme on Research for Integrating Services for the Maintenance of Autonomy (PRISMA 7). Data were gathered continuously on consecutive patients aged ≥ 70 years, 24 h per day, over a two-week period. A geriatric medicine-attuned frailty team (Health and Social Care Professionals along with an older adult physician) undertook a comprehensive geriatric assessment on screened participants to assign frailty status. This team was not aware of the frailty screening scores.

The fifth study by Piffer et al. (2020) [36] assessed ED physicians completing a version of the Triage Risk Screening Tool (TRST), labelled E-TRST, that assessed functional independence to accurately detect older adults with frailty who required a CGA. TRST consists of five questions on cognition, mobilisation, number of medications, recent ED or hospital admissions, and nursing feedback on specific ADL difficulties. The instrument was designed for ED nurses to highlight older adults with frailty who are at higher risk of adverse events. The study design was prospective. All adults aged ≥ 75 years admitted consecutively over a 1-year period to the ED of a French tertiary hospital were recruited. Those adults recruited were all assessed within 24 h, first by the E-TRST, which was completed by an emergency physician, then by a G-TRST version administered by a geriatrician, followed by the Short Emergency Geriatric Assessment (SEGA) as a gold standard comparison, which was completed by a geriatrician.

Positive responses to the first four questions elicited a score of 1. Each basic ADL score was divided into partially dependent (0.5 score) or fully dependent (1 score). Total scores range 0–5. TRST scores of ≥2 indicate frailty. SEGA evaluated thirteen variables: age, habitation, medication use, mood, self-reported standard of health, recent falls, diet, gait and balance, cognition, instrumental ADLs, incontinence, and meals. Scores ranged 0–2. The maximum SEGA tally is 26. In this study, SEGA > 8 indicated frailty.

The sixth study by McGuaran et al. (2020) [37] was undertaken in an emergency department in Ireland, screening 196 consecutive adults aged over 70 years over a 4-week period. Screening was undertaken during the weekdays, twenty-four hours a day, where possible. The study compared 3 and 4 variable versions of the Variable Indicator of Placement (VIP) with the CFS and PRISMA-7 to correctly identify frailty and predict adverse events including hospital admission, LOS, readmission (at 30–90 days), and deaths (over 3 months). The study team consisted of HSCPs, geriatric medicine trainees, nursing staff, and a geriatric medicine physician. This team undertook a comprehensive geriatric assessment of participants, blinded to the frailty screen scores. This was to determine true frailty status. Frailty cut-offs applied to screening tools were VIP-3 (score ≥ 2); VIP-4 (score ≥ 3); CFS (score ≥ 5); and PRISMA-7 (score ≥ 3).

**Table A1:** Table search strategy with number of citations according to each database.

Search Details	PubMed	Cinahl	Cochrane	Embase	Google Scholar	Trip	Total
**Search #1** FRAIL * OR PREFRAIL * OR PRE FRAIL *	18,514	8857	3967	24,609	N/A	11,797	43,135
**Search #2** TOOL * OR SCREEN * OR SCALE * OR SCORE * OR INSTRUMENT * OR MEASURE * OR INDEX * OR RISK OR PREDICTION *	949,076	376,975	1,529,902	1,400,342	N/A	1,801,459	6,057,754
**Search #3** “EMERGENCY DEPARTMENT” OR “EMERGENCY SERVICES” OR HOSPITAL *	929,624	305,742	309,120	1,272,570	N/A	96,115	2,913,171
**#1 AND #2**	10,199	3008	3967	18,819	N/A	60,259	79,322
**#1 AND #3**	8056	3008	1488	13,605	N/A	1427	27,584
**#1 AND # 2** **AND #3**	4972	3008	1480	10,684	63	1281	21,488
**Total citation count**							**21,488**

* refers to truncation, a symbol added to the end of the root of a word to instruct the database to search for all forms of a word. “” refers to quotation marks, a symbol that instructs the database to search for an exact phrase.

**Table A2:** Summary of frailty indicators measured in the included studies.

Tool	Reference	Self-Report of Fatigue	Function	Weight Loss	Mobility	Falls	Vision/ Hearing	Cognition	Nutrition	Social Support	Medication	Recent Hospitalization
**CFS**	McGauaran et al., 2020 [37] O’Caoimh et al., 2019 [10] Lague et al., 2018	+	+	-	+	-	-	+	-	-	-	-
**ISAR**	O’Caoimh et al., 2019 [10] Salvi et al., 2012 [8]	-	+	-	-	-	+	+	-	-	+	+
**TRST**	Piffer et al., 2020 [36]	-	+	-	+	+	-	+	-	-	+	+
**PRISMA-7**	McGauran et al., 2020 [37] O’Caoimh et al., 2019 [10]	-	+	-	+	-	-	-	-	+	-	-
**FRESH** **(4 Question version)**	Kajsa et al., 2016 [34]	+	+	-	-	+	-	-	-	-	-	-
**VIP**	McGauran et al., 2020 [37] O’Caoimh et al., 2019 [10]	-	+	-	-	-	-	-	-	+	-	-

Note: (-) refers to absence of frailty indicator; (+) refers to presence of frailty indicator.
